# A Case of Relapsing Peritoneal Dialysis-Associated Peritonitis by *Dokdonella koreensis*

**DOI:** 10.1155/2018/3820513

**Published:** 2018-07-05

**Authors:** Jamie Bee Xian Tan, Alvin Ren Kwang Tng, Htay Htay

**Affiliations:** ^1^Department of Microbiology, Singapore General Hospital, Outram Road, Singapore 169608; ^2^Department of Renal Medicine, Singapore General Hospital, Outram Road, Singapore 169608

## Abstract

Peritonitis is a common and serious complication of peritoneal dialysis (PD) with significant morbidity. We report the first case of relapsing *Dokdonella koreensis* peritonitis in a patient on peritoneal dialysis. A 63-year-old Chinese man, with history of renal failure on continuous ambulatory peritoneal dialysis, presented with cloudy peritoneal effluent and abdominal pain. There was no sign or symptom suggestive of exit-site/tunnel tract infection. Peritoneal effluent cultures yielded *Dokdonella koreensis* which was initially misidentified as *Weeksella virosa* and *Brevundimonas* species by the API® 20 NE and VITEK® 2 GN ID card, respectively. He was treated with intraperitoneal amikacin, but the infection relapsed within a few days upon completing each antibiotic course. He eventually required removal of catheter and was transferred to hemodialysis. Infections due to unusual organisms may pose a diagnostic issue as currently available commercial tests will not be able to identify them. There is a role for using 16S rRNA sequencing to help identify these organisms and guide patient management.

## 1. Introduction

Peritoneal dialysis-associated peritonitis is a common and serious complication that can lead to technique failure and death. It is usually caused by bacteria whereas a small percentage is attributable to fungi [1–8]. *Dokdonella koreensis* is a Gram-negative bacilli that was first isolated from the soil and has been reported previously in patients with catheter-related bloodstream infections. We report herein the first case of relapsing PD-associated peritonitis due to *Dokdonella koreensis* over a three-month period in a patient with end-stage renal failure.

## 2. Case Report

A 63-year-old Chinese male with end-stage renal failure secondary to reflux nephropathy had been undergoing continuous ambulatory PD for the past two years. He had an episode of PD catheter exit-site infection with *Staphylococcus aureus* and *Corynebacterium* species a year ago that was treated with antibiotics. He first presented to the PD centre with a two-day history of cloudy peritoneal effluent and mild intermittent colicky right-sided abdominal pain. He was afebrile and did not report any vomiting or diarrhoea. He frequently performs gardening but did not recall any episode of sterile technique breach while performing the peritoneal dialysis exchanges.

On physical examination, the PD catheter exit-site was clean without any discharge expressed. There was also no tunnel tract tenderness and overlying erythema. The white cell count of the peritoneal effluent was 1.6 × 10^9^/L with 63% neutrophils. Gram stain showed polymorphonuclear leukocytes 2+ but no organisms. Peritoneal effluent culture yielded *Weeksella virosa* that was sensitive to amikacin. The patient was managed as an outpatient with intraperitoneal (IP) vancomycin and amikacin initially as per the institution protocol which was subsequently adjusted to complete a two-week course of IP amikacin. In the institution, peritonitis caused by single Gram-negative organism which improves with antibiotics are treated for two weeks as recent studies using registry data reported that the cure rate of peritonitis did not differ between patients who were treated for ≤two weeks and those who were treated for >two weeks [9, 10]. The peritoneal effluent white cell count normalized within four days of treatment.

Ten days after stopping amikacin, however, the patient presented to the PD centre again with cloudy peritoneal effluent and abdominal pain. The PD catheter exit-site was clean. Peritoneal effluent white cell count had risen to 6.9 × 10^9^/L with 74% neutrophils. Gram stain of the peritoneal effluent again showed polymorphonuclear leukocytes 3+ but no organisms, whereas culture yielded *Brevundimonas* species that was sensitive to amikacin. He was diagnosed with recurrent peritonitis, which was defined as an episode that occurs within 4 weeks of completion of therapy of a prior episode but with a different organism [11]. He was started on another course of IP amikacin with resolution of symptoms and the peritoneal effluent white cell count decreasing to 0.3 × 10^9^/L within three days.

Two days following the completion of the second course of amikacin, the patient presented yet again with cloudy peritoneal effluent and right-sided colicky abdominal pain. He admitted to a breach in the sterile technique. There was no fever and no pus at the PD catheter exit-site. The decision was then made to admit him for treatment and investigation of the recurrent peritonitis as an inpatient. On admission, the white blood cell count was 7.34 × 10^9^/L, C-reactive protein was elevated at 77.3 mg/L, and the peritoneal effluent white cell count was 2.3 × 10^9^/L with 82% neutrophils. Computed tomography of the abdomen and pelvis did not show any intra-abdominal abscess or collection. The repeat culture of the peritoneal effluent yielded *Dokdonella koreensis* that was sensitive to amikacin. Cultures for fungi and acid-fast bacilli were negative. He was advised to remove the PD catheter and convert temporarily to hemodialysis in view of the recurrent peritonitis, but he declined catheter removal despite being informed of the consequences of prolonged attempt to treat recurrent peritonitis without catheter removal, which included extended hospitalization, damage to the peritoneal membrane, increased risk of fungal peritonitis, and mortality. He responded to IP amikacin with the peritoneal effluent white cell count normalizing within five days. The sterile technique was reinforced to him, and he was discharged to complete the course of IP amikacin.

Three days after completing the third course of amikacin, the patient was admitted again with cloudy peritoneal effluent and abdominal pain. He claimed adherence to the aseptic technique for dialysis exchange and again declined catheter removal. Peritoneal effluent culture grew *Dokdonella koreensis* that was sensitive to amikacin. The peritoneal effluent white cell count normalized within six days of commencing IP amikacin for the relapsing peritonitis, which was defined as an episode that occurs within 4 weeks of completion of therapy of a prior episode with the same organism or one sterile episode [11].

After hospital discharge, the patient was admitted yet again within two weeks for a traumatic hip fracture and was found at the same time to have raised peritoneal effluent white cell count of 1.6 × 10^9^/L. Repeat cultures this time had no bacterial growth. It was during this inpatient admission and about three months after his initial presentation to the clinic that he finally agreed to remove the PD catheter and temporarily convert to hemodialysis. He remains well to this date, and his long-term dialysis options will be revisited in a few months' time.

### 2.1. Investigations

**Figure 1:**
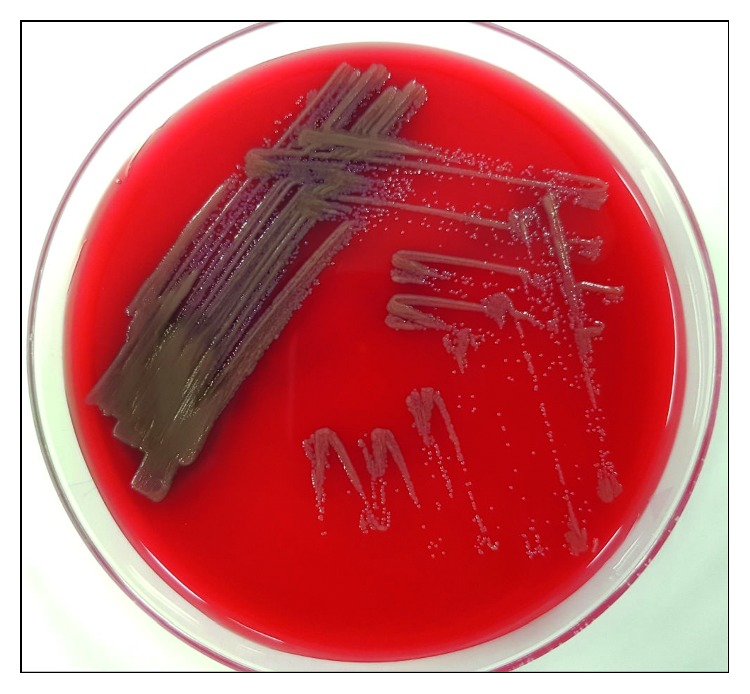
Small yellow colonies on 5% sheep blood agar after 48 hours of incubation.

**Figure 2:**
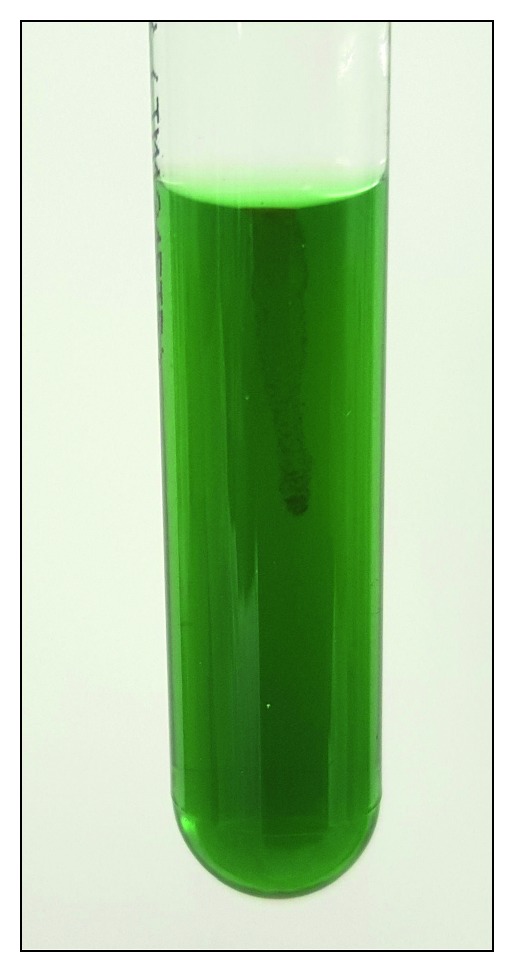
*Dokdonella koreensis* was nonmotile when tested in motility tube media.

Peritoneal effluent was first inoculated into a blood culture bottle which was incubated for 24 hours at 35°C in O_2_ before it was plated onto 5% sheep blood agar. After 48 hours of incubation at 35°C in 5% CO_2_, small flat yellow circular colonies were seen (Figure 1). Gram stain of these colonies showed Gram-negative bacilli. The organism was an obligate aerobe. It was oxidase positive, catalase positive, and nonmotile (Figure 2). No reliable identification was given via matrix-assisted laser desorption ionization—time of flight mass spectrometry (MALDI-TOF MS), but it was identified as *Weeksella virosa* by the API 20 NE and *Brevundimonas* species by the VITEK 2 GN ID card. In view of the discordant bacterial identification results from the API 20 NE and the VITEK 2 GN ID card, 16S rRNA gene sequencing was performed which subsequently identified the organism as *Dokdonella koreensis*. Antimicrobial susceptibility testing was done using the Etest® which found the organism susceptible to various antibiotics such as amikacin, levofloxacin, ciprofloxacin, piperacillin-tazobactam, cefepime, and meropenem (minimum inhibitory concentration values of 8 g/L, 0.125 g/L, 0.5 g/L, 0.5 g/L, 0.25 g/L, and 0.032 g/L, resp.) [12].

## 3. Discussion

*Dokdonella koreensis* is a non-spore-forming Gram-negative bacilli. It was first isolated in 2006 from the soil of Dokdo, an island located east of Korea [13]. Reports of *Dokdonella* species causing human infections are rare. Two case reports in 2007 and 2014 described the isolation of *Dokdonella koreensis* from the blood culture of two acute myeloid leukemia patients who had catheter-related bloodstream infection that resolved with intravenous antibiotics and catheter change/removal [14, 15]. Similar to our case, bacterial identification was only possible through 16S rRNA gene sequencing as it could not be identified using routine phenotypic methods.

To our knowledge, our patient represents the first documented case of *D. koreensis* peritonitis in a patient undergoing continuous ambulatory peritoneal dialysis. Out of nine cultures of the peritoneal effluent that were sent over a period of three months, five were positive for *D. koreensis*. Peritoneal dialysis-related peritonitis can be due to touch contamination [16, 17], exit-site/tunnel tract infection, or an underlying gastrointestinal pathology. Our patient did not have any signs, symptoms, or positive radiological findings suggestive of an exit-site/tunnel tract infection or underlying gastrointestinal pathology. As *Dokdonella* species are usually isolated from the soil [13], we postulated that our patient likely acquired the infection through contamination given that he performed the peritoneal dialysis exchange himself at home with an episode of sterile technique breach and also had a history of frequent gardening.

Another thing to note in our case was the relapsing peritonitis despite treatment with multiple courses of intraperitoneal antibiotics based on the sensitivity result. As the *D. koreensis* isolates that were repeatedly cultured from the peritoneal effluent cultures remained susceptible in vitro to amikacin, it is unlikely that treatment failure was due to the development of resistance. A possible reason for treatment failure in this case is biofilm formation on the PD catheter affecting drug penetration, [18–22] thereby leading to treatment failure. The recommended treatment for relapsing peritonitis is catheter removal [11].

In summary, this report describes the first case of relapsing peritoneal dialysis-related peritonitis caused by *D. koreensis* and the importance of using 16S rRNA sequencing to help identify unusual organisms that may have an emerging role in causing human infections.
